# Analyses of chondrogenic induction of adipose mesenchymal stem cells by combined co-stimulation mediated by adenoviral gene transfer

**DOI:** 10.1186/ar4260

**Published:** 2013-07-30

**Authors:** Idalia Garza-Veloz, Viktor J Romero-Diaz, Margarita L Martinez-Fierro, Ivan A Marino-Martinez, Manuel Gonzalez-Rodriguez, Herminia G Martinez-Rodriguez, Marcela A Espinoza-Juarez, Dante A Bernal-Garza, Rocio Ortiz-Lopez, Augusto Rojas-Martinez

**Affiliations:** 1 Departamento de Bioquimica y Medicina Molecular, Facultad de Medicina, Universidad Autonoma de Nuevo Leon, Monterrey C.P. 64460, Mexico; 2 Laboratorio de Medicina Molecular, Unidad Academica de Medicina Humana y Ciencias de la Salud, Universidad Autonoma de Zacatecas, Zacatecas, C.P 98160 Mexico; 3 Departamento de Histologia, Facultad de Medicina, Universidad Autonoma de Nuevo Leon, Monterrey C.P. 64460, Mexico; 4 Unidad de Terapia Genica y Celular, Centro de Investigación y Desarrollo en Ciencias de la Salud, Universidad Autonoma de Nuevo Leon, Monterrey C.P. 64460, Mexico

## Abstract

**Introduction:**

Adipose-derived stem cells (ASCs) have the potential to differentiate into cartilage under stimulation with some reported growth and transcriptional factors, which may constitute an alternative for cartilage replacement approaches. In this study, we analyzed the *in vitro *chondrogenesis of ASCs transduced with adenoviral vectors encoding insulin-like growth factor-1 (IGF-1), transforming growth factor beta-1 (TGF-β1), fibroblast growth factor-2 (FGF-2), and sex-determining region Y-box 9 (SOX9) either alone or in combinations.

**Methods:**

Aggregate cultures of characterized ovine ASCs were transduced with 100 multiplicity of infections of Ad.IGF-1, Ad.TGF-β1, Ad.FGF-2, and Ad.SOX9 alone or in combination. These were harvested at various time points for detection of cartilage-specific genes expression by quantitative real-time PCR or after 14 and 28 days for histologic and biochemical analyses detecting proteoglycans, collagens (II, I and X), and total sulfated glycosaminoglycan and collagen content, respectively.

**Results:**

Expression analyses showed that co-expression of IGF-1 and FGF-2 resulted in higher significant expression levels of aggrecan, biglycan, cartilage matrix, proteoglycan, and collagen II (all *P *≤0.001 at 28 days). Aggregates co-transduced with Ad.IGF-1/Ad.FGF-2 showed a selective expression of proteoglycans and collagen II, with limited expression of collagens I and × demonstrated by histological analyses, and had significantly greater glycosaminoglycan and collagen production than the positive control (*P *≤0.001). Western blot analyses for this combination also demonstrated increased expression of collagen II, while expression of collagens I and × was undetectable and limited, respectively.

**Conclusion:**

Combined overexpression of IGF-1/FGF-2 within ASCs enhances their chondrogenic differentiation inducing the expression of chondrogenic markers, suggesting that this combination is more beneficial than the other factors tested for the development of cell-based therapies for cartilage repair.

## Introduction

Articular cartilage is a highly specialized connective tissue with a unique architecture that enables almost frictionless articulation of joint surfaces and the ability to absorb mechanical stress. Although remarkably durable, articular cartilage has a restricted capacity for intrinsic regeneration, and even minor injuries may lead to progressive damage and subsequent join degeneration [1,2]. Adult mesenchymal stem cells (MSCs) present a viable alternative for primary differentiated chondrocytes that must be isolated from very limited sources of cells and are difficult to expand *ex vivo *[3,4]. Adipose-derived stem cells (ASCs) have been shown to possess multilineage differentiation potential into osteogenic, chondrogenic, adipogenic, myogenic, neurogenic, and endothelial cells in the presence of lineage-specific induction factors, and have been characterized extensively for chondrogenesis [5,6]. ASCs are abundant in fat tissues and are relatively easy to obtain and expand in culture. These cells exhibit low rates of senescence, even after nine or more passages [7,8].

*In vitro *chondrogenesis of ASCs is a finely regulated process that requires appropriate expanded monolayer conditions and subsequent high-density culture in specific media supplements and growth factor-containing medium. Potentially useful growth factors are members of the transforming growth factor beta (TGF-β) superfamily, including TGF-β1, TGF-β2 and TGF-β3, several bone morphogenic proteins, insulin-like growth factor-1 (IGF-1), fibroblast growth factors, and epidermal growth factor, among others [9]. Numerous studies have shown that co-administration of TGF-β1 and IGF-1 efficiently stimulates chondrogenic differentiation and increase matrix synthesis of chondrogenic cells [10-12]. *In vivo *co-administration of IGF-1 and FGF-2 has also been reported to accelerate articular cartilage repair [13], while administration of FGF-2 in the presence of TGF-β1 significantly enhances cell proliferation, which results in increased neocartilage formation at later stages [14].

Another class of biologics that promote chondrogenesis are the transcription factors sex-determining region Y-box 9 (SOX9) and related L-SOX5 and SOX6. These factors have been identified as essential factors for chondrocyte differentiation and cartilage formation [15]. However, the short half-lives of recombinant proteins, and a lack of effective delivery methods for intracellular signaling, challenge the clinical uses of these factors.

Gene transfer offers an alternative approach to protein delivery that may satisfactorily overcome the limitations of conventional methods [9,16,17]. Viral vectors can be used to deliver cDNAs that code for therapeutic proteins to specific target cells, and the genetically modified cell is converted in a biofactory for protein production [18]. Sustained protein synthesis can be concentrated at the site of injury by *in situ *gene delivery with minimal collateral exposure of nontarget tissues [16].

The effectiveness of several growth-factor combinations for chondrogenic differentiation of ASCs is still unclear. Methods to effectively stimulate proliferation and chondrogenic differentiation of ASCs are needed to further develop the use of these cells for cartilage repair. The effects of expression of adenoviral vectors carrying IGF-1, TGF-β1, FGF-2 and SOX9 cDNAs on chondrogenesis of primary ASCs *in vitro*, using single vectors and/or their combinations, were also evaluated in this study.

## Materials and methods

### Preparation of recombinant adenoviral vectors

First-generation, E1, E3-deleted, serotype 5 adenoviral vectors carrying the cDNAs for GFP, human IGF-1, human TGF-β1, human FGF-2, and human SOX9 were constructed using the method of Luo and colleagues [19]. The resulting vectors were designated Ad.GFP, Ad.IGF-1, Ad.TGF-β1, Ad.FGF-2, and Ad.SOX9, respectively. To generate high-titer preparations, the recombinant vectors were amplified in HEK-293 cells and purified over three successive cesium chloride gradients. Following dialysis against 10 mM Tris-hydrochloric acid, pH 7.4, 150 mM sodium chloride, 10 mM magnesium chloride, and 4% sucrose, the preparations were aliquoted and stored at -80°C. Viral titers were estimated by optical density (at 260 nm) and median tissue culture infectious dose methods. Using these methods, preparations of 10^7 ^to 10^9 ^plaque-forming units/ml were obtained

### Adipose-derived stem cell isolation, culture and characterization

The protocol involving research in animals was approved by the UANL School of Medicine & University Hospital Institutional Review Board (reference number: BI12-002) and experiments were conducted following the Mexican ordinances for the treatment of experimental animals (Norma Oficial Mexicana 062-ZOO-1999).

ASCs were harvested from the adipose tissue of one 6-month-old *Ovis aries *weighing 37.4785 lb, and 0.5 g adipose tissue biopsy specimens were digested with 800 µl collagenase I (180 U/ml) solution using the protocol of Dubois and colleagues [20]. The collected cells were pelleted using centrifugation at 1,500 rpm for 10 minutes, and resuspended in DMEM containing 10% fetal bovine serum (FBS) and 1% penicillin/streptomycin/amphotericin B (all Invitrogen, Carlsbad, CA, USA). The cells were plated in a 75 cm^2 ^tissue culture flask (Falcon, Beckton Dickinson Labware, Franklin Lakes, NJ, USA). Nonadherent cells were removed after 3 days; the remaining attached cells were washed with PBS and cultured in DMEM with 10% FBS at 37°C, 5% CO_2 _with medium changes every 3 days. After 10 to 15 days, adherent colonies of cells were trypsinized and replated in several 75 cm^2 ^tissue culture flasks, six-well or 96-well plates depending on the procedure.

To confirm the ASC phenotype, cell cultures were characterized through immunophenotype and RT-PCR. Flow cytometry was performed on a FACScan argon laser cytometer (Becton Dickson, San Jose, CA, USA). Cells were harvested in 0.25% trypsin/ethylenediaminetetraacetic acid and fixed for 30 minutes in ice-cold 2% formaldehyde. Following fixation, cells were washed in flow cytometry buffer (1 × PBS, 2% FBS, 0.2% Tween-20). Cell aliquots (1 ×10^6 ^cells) were incubated in flow cytometry buffer containing the following mAbs: anti-CD271-PE, anti-CD45-FITC and anti-mesenchymal stromal cell antigen-1-APC (all AbD Serotec, Kidlington, UK). In addition, RNA was isolated from primary ASC cultures according to the TRIzol^® ^Reagent protocol (Invitrogen). cDNA was synthesized from total RNA using the SuperScript™ III First-Strand Synthesis SuperMix and random hexamers (Invitrogen). One hundred nanograms of cDNA synthesized were used as templates for PCR amplification in a 25 µl reaction volume using *Taq *DNA polymerase (Promega, Madison, WI, USA) and 500 nM gene-specific primers. Amplifications were performed for 35 cycles, and RT-PCR products were visualized on 2% agarose gels containing 0.1 µg/ml ethidium bromide. The primer sequences and product sizes for CD34, CD73, CD90, CD105, CD166, CD45, CD117, CD271, CD14, and glyceraldehyde-3-phosphate dehydrogenase (GAPDH) are listed in Additional file 1.

### Cell viability assay

ASCs were seeded into 96-well plates and grown to 80% confluence, generating approximately 2.6 × 10^4 ^cells/well. Individual wells of cells, in triplicate, were transduced in 100 µl serum-free DMEM for 2 hours with decreasing doses (1,000, 100, 10 and 1 multiplicity of infections (MOIs)) of individual Ad.GFP, Ad.IGF-1, Ad.TGF-β1, Ad.FGF-2, and Ad.SOX9 vectors or combinations (Ad.IGF-1/Ad.TGF-β1, Ad.IGF-1/Ad.FGF-2, Ad.IGF-1/Ad.TGF-β1/Ad.SOX9, and Ad.IGF-1/Ad.FGF-2/Ad.SOX9). Following transduction, the culture fluids were aspirated and replaced with 200 µl DMEM containing 2% FBS and 1% penicillin/streptomycin/amphotericin B. In parallel, control nontransduced cultures were maintained in the same medium. Cells were incubated at 37°C, 5% CO_2 _for 10 days, and then viability was measured according to the Alamar Blue^® ^protocol (Invitrogen).

### Adenoviral transduction of adipose-derived stem cells in monolayers

Following the initial plating, the adherent cultures of ASCs were seeded into six-well plates and grown to 80% confluence, generating approximately 7.6 × 10^5 ^cells/well. Individual wells of cells, in triplicate, were transduced in 800 µl serum-free DMEM for 2 hours with 100 MOIs of Ad.IGF-1, Ad.TGF-β1, Ad.FGF-2 and Ad.SOX9 alone or in combination (Ad.IGF-1/Ad.TGF-β1, Ad.IGF-1/Ad.FGF-2, Ad.IGF-1/Ad.TGF-β1/Ad.SOX9 and Ad.IGF-1/Ad.FGF-2/Ad.SOX9), using 50 + 50 MOIs by two vectors or 33.3 + 33.3 + 33.3 MOIs by three vectors (100 MOIs together), respectively. Negative control cultures were similarly transduced with Ad.GFP.

Following transduction, the culture fluids were aspirated and replaced with 2 ml DMEM containing 25 mM glucose, 6.25 µg/ml insulin-transferrin-sodium selenite, 5.33 µg/ml linoleic acid, 1.25 mg/ml BSA, 100 nM dexamethasone, 50 µg/ml L-ascorbic-2-phosphate, 2 mM sodium pyruvate, 40 µg/ml L-proline (all Sigma-Aldrich, St Louis, MO, USA), 10% FBS and 1% penicillin/streptomycin/amphotericin B. In parallel, non-transduced cultures (positive control), were replaced with 2 ml HyClone AdvanceSTEM Chondrogenic Differentiation Medium (Thermo Scientific, Rockford, IL, USA). The cells were cultured at 37°C, 5% CO_2 _and began to form spherical aggregates after 3 days of culture, excepting the negative control that was maintained in a monolayer. Media were changed every 3 days. Cultures were harvested at various time points for quantitative real time (qRT)-PCR analyses or after 14 days for histologic and biochemical analyses. Ad.GFP transduced cultures were viewed for fluorescence at 72 hours following transduction.

### Quantitative real time PCR assay

qRT-PCR was used to evaluate quantitatively transcription both transgene expression and cartilage-specific genes following transduction of ASCs with 100 MOIs of Ad.IGF-1, Ad.TGF-β1, Ad.FGF-2 and Ad.SOX9 alone or in combination (Ad.IGF-1/Ad.TGF-β1, Ad.IGF-1/Ad.FGF-2, Ad.IGF-1/Ad.TGF-β1/Ad.SOX9 and Ad.IGF-1/Ad.FGF-2/Ad.SOX9), respectively. Total RNA was isolated from each triplicate group of ASCs grown in monolayer or aggregates cultured per time points (0, 3, 14, and 28 days), using TRIzol^® ^Reagent (Invitrogen). cDNA was synthesized from total RNA using SuperScript™ III First-Strand Synthesis SuperMix and random hexamers (Invitrogen).

qRT-PCR was performed using a CFX96 real-time PCR detection system (Bio-Rad, Hercules, CA, USA) in 96-well PCR plates. Twenty nanograms of synthesized cDNA were used as templates for qRT-PCR amplification in a 15 µl final reaction volume using 1 × iQ™ SYBR^® ^Green Supermix (Bio-Rad), and 500 nM gene-specific primers, which were designed based on the respective GenBank sequence for the examined gene. Amplifications were performed with the following thermal cycle program: predenaturation for 10 minutes at 95°C, PCR amplification for 40 cycles of denaturizing for 15 seconds at 95°C, and annealing for 1 minute at 60°C. Cycle series were followed by melt-curve analyses to check the specificity of the reaction. Sequences and product sizes of forward and reverse primers for aggrecan (AGC), biglycan (BGC), cartilage matrix (CM), collagen I (COL I), collagen II (COL II), collagen × (COL X), proteoglycan (PGC), IGF-1, TGF-β1, FGF-2, SOX9, and GAPDH are listed in Additional file 1. The efficiency and specificity of each primer set was confirmed with standard curve and melting profile evaluation; the efficiency of amplification relative to GAPDH gene was confirmed with standard curve; all this accords with a standardization reported before [21].

### Aggregate culture and protein expression

Following the initial plating, the adherent cultures of ASCs were seeded into six-well plates and grown to 80% confluence, generating approximately 7.6 × 10^5 ^cells/well. Individual wells of cells, in triplicate, were transduced in 800 µl serum-free DMEM for 2 hours with 100 MOIs of Ad.IGF-1, and Ad.FGF-2 alone or in combination. Following transduction, the culture fluids were aspirated and replaced with 2 ml DMEM containing 25 mM glucose, 6.25 µg/ml insulin-transferrin-sodium selenite, 5.33 µg/ml linoleic acid, 1.25 mg/ml BSA, 100 nM dexamethasone, 50 µg/ml L-ascorbic-2-phosphate, 2 mM sodium pyruvate, 40 µg/ml L-proline (all Sigma-Aldrich, St Louis, MO, USA), 10% FBS and 1% penicillin/streptomycin/amphotericin B. The cells were cultured at 37°C, 5% CO_2 _and began to form spherical aggregates after 3 days of culture. Media were collected and changed at 3, 7, 14, and 21 days, and the aggregates were harvested at 14 and 28 days for ELISA analyses for the respective growth factors using the appropriate commercially available ELISA kits (Abcam Inc., Cambridge, MA, USA) for human IGF-1 and FGF-2.

### Biochemical analysis

Three aggregates per group, cultured for 28 days, were digested for 18 hours at 65°C by incubating them in 1 ml papain solution containing 125 µg/ml papain with 5 mM L-cysteine-HCl and 5 mM ethylenediaminetetraacetic acid in 100 mM sodium phosphate monobasic (pH 6.2). The total sulfated glycosaminoglycan (GAG) content was determined using shark chondroitin sulfate as the standard and measuring the sample content with the 1,9-dimethylmethylene blue assay. The total collagen content was determined by measuring the hydroxyproline content of the aggregates after acid hydrolysis and reaction with *p*-dimethylaminobenzaldehyde and chloramine-T, using 0.134 as the ratio of hydroxyproline to collagen (all Sigma-Aldrich). Both the total collagen content and GAG content were normalized to the total DNA content, which was measured fluorometrically using the Hoechst 33258 dye (bisbenzimide) DNA quantitation kit according to the manufacturer's protocol (excitation wavelength, 485 nm; emission wavelength, 535 nm; Bio-Rad). The DNA concentration was determined from a standard curve of calf thymus DNA (Bio-Rad).

### Histological and immunohistochemical analysis

Before tissue processing, representative aggregates of each group were photographed using a digital camera (Model C653; Kodak, Rochester, NY, USA). For histological analyses, aggregates cultured for 14 and 28 days were embedded in Tissue-Tek^® ^O.C.T™ Compound (Sakura Finetek, Torrance, CA, USA) to ease handling, and then sectioned to 10 µm in thickness at -20°C using a Tissue-Tek cryostat (Model 4553; Miles Inc., Elkhart, IN, USA). Representative sections were stained using toluidine blue for the detection of matrix proteoglycan, and safranine-O/fast green staining for the detection of accumulation of sulfated proteoglycans (all Sigma-Aldrich).

For immunohistochemistry, sections of aggregates cultured for 28 days prepared as described above were rinsed with PBS and treated sequentially with 30% (vol/vol) H_2_O_2_/methanol at a ratio of 1:9 for 10 minutes, and 0.15% TritonX-100 in 1 × PBS for 10 minutes. Sections were then blocked with 5% BSA in PBS for 30 minutes. Afterwards, the sections were incubated overnight at 4°C with mouse monoclonal anti-COL I and anti-COL II primary antibodies (all Abcam Inc.) and mouse monoclonal anti-COL × (Sigma-Aldrich) diluted in 1% BSA in PBS. After three PBS washes to remove unbound primary antibody, sections were incubated with a biotinylated secondary antibody against mouse IgG for 1 hour and peroxidase-conjugated streptavidin solution for 30 minutes at room temperature (both DakoCytomation, Carpinteria, CA, USA). The slides were washed again and mounted in Fluoromount-G™ (SouthernBiotech, Birmingham, AL, USA), and coverslipped for microscopic observation (Model E600; Nikon Corporation, Tokyo, Japan). The negative control consisted of monolayer ASCs cultured in incomplete chondrogenic medium, fixed, and immunostained *in situ*. For each experiment described, three replicates were performed, with three aggregates for each group.

### Western blot analysis and densitometry

Approximately 3 × 10^6 ^ASCs per 75 cm^2 ^plate were transduced with Ad.IGF-1/Ad.FGF-2 (50 MOIs each), were nontransduced but stimulated with HyClone AdvanceSTEM Chondrogenic Differentiation Medium (Thermo Scientific) (positive control), and were not transduced and non-stimulated ASCs grown in DMEM (negative control). Total protein extract was obtained at 28 days post transduction. COL I, COL II, and COL × were detected by western blot analysis at day 28. Briefly, 50 µg total protein extract in Laemmli buffer was subjected to 10% SDS-PAGE and transferred to a nitrocellulose membrane; blocking was performed with nonfat milk 5% in Tris-buffered saline with Tween GAPDH was detected as a sample loading control using a primary rabbit polyclonal anti-human GAPDH (dilution 1:5,000; Santa Cruz Biotechnology Inc., Santa Cruz, CA, USA) and a horseradish peroxidase conjugated with goat anti-rabbit IgG antibodies (dilution 1:5,000; Santa Cruz Biotechnology Inc.). Primary rabbit polyclonal anti-human type I, II and × collagen (dilution 1:3,000; Abcam) were used to detect COL I, COL II, and COL X, respectively. Bound antibodies were detected with horseradish peroxidase conjugated with goat anti-rabbit IgG antibodies (1:5,000; Abcam). A densitometric analysis using GAPDH expression for assay normalization was performed (Phoretix 1D software; TotalLab Ltd, Newcastle, UK).

### Statistical analysis

Data from the cell viability assay, qRT-PCR and biochemical assays were analyzed for statistical significance between two comparative specimens by a Student *t *test or Mann-Whitney U test according to data distribution normality, using SigmaPlot v11.0 (Systat Software Inc., San Jose, CA, USA). Data are presented as mean ± standard deviation. Differences were considered of statistical significance when *P *<0.05.

## Results

### Phenotypic characterization of adipose-derived stem cells

First-passage cells were characterized through stem cell marker detection using immunophenotype by flow cytometry and RT-PCR. The immunophenotype showed that the expression of CD271, mesenchymal stromal cell antigen-1 and CD45 were 85.82%, 95.55% and 36.78%, respectively. RT-PCR showed amplification for CD73, CD90, CD14, CD166, CD105, CD271, and GAPDH, and no amplification for CD34, CD45, and CD117. This expression profile is typical for ASCs except for CD45 and CD14. Phenotypic characterizations are summarized in Table 1[22,23]. In general, these results demonstrated successful ASC isolation.

**Table 1:** Phenotypic characterization of adipose-derived stem cells through immunophenotype and RT-PCR

Analyzed marker	Immunophenotype^a^	RT-PCR^b^
Positive^c^		
CD73	NT	++
CD90	NT	++
CD105	NT	++
CD166	NT	++
CD271	++	++
MSCA	++	NT
Negative^c^		
CD14	NT	+
CD34	NT	-
CD45	+	-
CD117	NT	-
GAPDH^d^	NT	++

^a^NT, not tested; +, positive (≤50%); ++, positive (≥85%). ^b^NT, not tested; -, negative; +, positive (faint band); ++, positive (intense band). ^c^Expected result of surface antigen expression on mesenchymal stem cells according to the literature [22,23]. ^d^Housekeeping gene.

### Cell viability and transduction efficiency of adipose-derived stem cells with adenoviral vectors

To determine the adenoviral concentration at which adherent ASCs can be genetically modified with one, two or three anabolic transgenes in high-density culture without compromising their viability, first-passage monolayer cultures were transduced with recombinant Ad.IGF-1, Ad.TGF-β1, Ad.FGF-2, or Ad.SOX9 alone or in combination at total viral doses of 1, 10, 100 or 1,000 MOIs, as indicated in Materials and methods; vector combinations were performed at 1:1 and 1:1:1 ratios for combination of two and three vectors, respectively. Control groups consisted of naive and transduced ASC cultures with equivalent doses of adenoviral vectors encoding Ad.GFP. First-passage monolayer cultures were also transduced with the same increasing amounts of Ad.GFP to provide a relative comparison for transduction efficiency. After 72 hours and consistent with the ASC cultures, GFP-positive cells appeared with the typical fibroblast-like morphology. One hundred MOIs were selected to transduce ASCs due to the high level of transduction (>90%) and >80% cell viability (see Additional file 2).

### Chondrogenic differentiation of adipose-derived stem cells after adenoviral delivery of IGF-1, TGF-β1, FGF-2 and SOX9 alone or in combination

**Figure 1:**
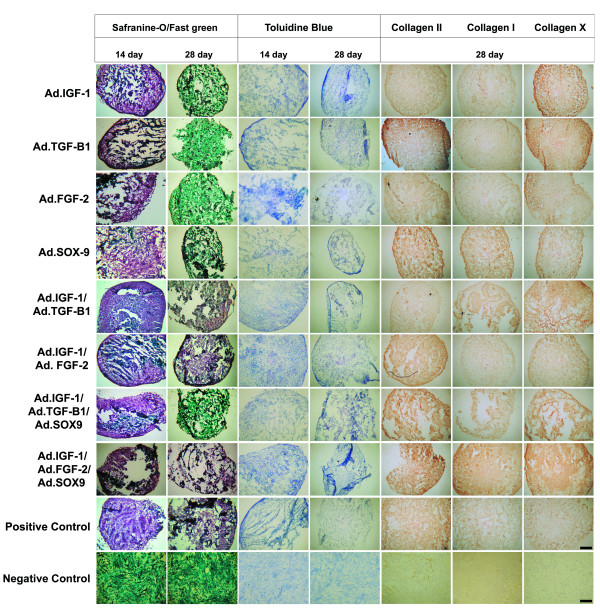
**Gene expression in genetically modified adipose-derived stem cells aggregate cultures**. Adipose-derived stem cells **(**ASCs) were transduced with 100 multiplicity of infections of respective adenoviral vectors as indicated, cultured into aggregates, and maintained in a defined serum-free medium for 3, 7, 14, 21 or 28 days. For each treatment group and time point indicated, RNA was extracted from three aggregates, and both expression of **(A) **tranduced genes (3, 7, 14, 21 and 28 days) and **(B,C,D,E,F,G,H) **the cartilage-specific marker genes aggrecan (AGC), biglycan (BGC), cartilage matrix (CM), and proteoglycan (PGC), collagen (COL) I, COL II, COL X, (3, 14, and 28 days) were determined by quantitative real time (qRT)-PCR. RNA isolated from ASCs differentiated by a commercial established medium and RNA extracted immediately from ASCs newly transduced (time 0) were used as comparative controls. The primer sequences, product sizes, and annealing temperatures for qRT-PCR are listed in Additional file 1. The expression level of each targeted gene was normalized to the housekeeping gene GAPDH. Values are expressed as the fold induction of means ± standard deviations of normalized expression levels. Statistical differences between groups and positive control were analyzed using a *t *test; *differences were considered significant when *P *<0.05. FGF-2, fibroblast growth factor-2; IGF-1, insulin-like growth factor-1; SOX9, sex-determining region Y-box 9; TGFβ, transforming growth factor beta.

To determine the relative level of transgene expressed, parallel cultures of ASCs were transduced with 100 MOIs of Ad.IGF-1, Ad.TGFβ-1, Ad.FGF-2, and Ad.SOX9, both in single and combined transductions. For each experimental group, transgene expression was decreasing through time (3, 7, 14, 21 and 28 days; Figure 1A). Because primary ASCs were shown to be able of sustained expression of different anabolic transgenes after adenoviral-mediated transduction (see Additional file 3), the effects of growth factor co-expression on *in vitro *chondrogenesis of ASC aggregates were analyzed. Second-passage monolayer cultures of ASCs (7.6 × 10^5 ^ASCs) were transduced in triplicate with 100 MOIs of Ad.IGF-1, Ad.TGFβ-1, Ad.FGF-2, and Ad.SOX9, both in single and combined transductions. Following transduction, the culture fluids were aspirated and replaced with a defined supplemented medium. The cells began to form spherical aggregates after 3 days of culture; they were maintained for 28 days, being harvested at 14 and 28 days to be analyzed.

**Figure 2:**
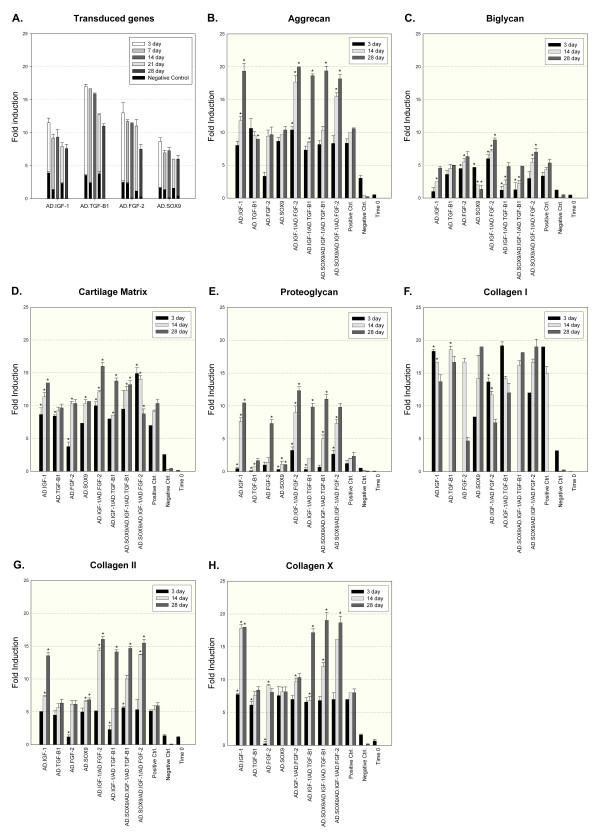
**Chondrogenesis of adipose-derived stem cells following adenoviral-mediated gene transfer**. Monolayer cultures of adipose-derived stem cells **(**ASCs) were transduced with 100 multiplicity of infections of Ad.IGF-1, Ad.TGF-β1, Ad.FGF-2 and Ad.SOX9 single or in combination; following aggregate formation, the aggregates were cultured for 28 days and harvested at 14 and 28 days. Non-transduced positive and negative control ASCs were cultured in parallel. Safranine-O and Toluidine blue stainings were used for the detection of matrix proteoglycan in representative aggregate sections, and immunostaining was used for the presence of collagens; predominant collagen (COL) II indicates a chondrocyte-like phenotype, predominant COL I indicates a fibrous matrix, and predominant COL × indicates hypertrophic chondrocytes undergoing terminal differentiation. Negative control for the stainings corresponds to ASCs cultured in incomplete chondrogenic medium. All 100 × magnification, bar = 200 µm. FGF-2, fibroblast growth factor-2; IGF-1, insulin-like growth factor-1; SOX9, sex-determining region Y-box 9; TGFβ, transforming growth factor beta.

Histological examination indicated evidence of transgene-induced chondrogenesis of the ASCs. Aggregates receiving Ad.FGF-2 together with Ad.IGF-1 had greater chondrogenic response than aggregates receiving the adenovirus alone (Ad.IGF-1, Ad.TGF-β1, Ad.FGF-2, Ad.SOX9) or in other combinations (Ad.IGF-1/Ad.TGF-β1, Ad.IGF-1/Ad.TGF-β1/Ad.SOX9, Ad.IGF-1/Ad.FGF-2/Ad.SOX9). This response was demonstrated by the production of COL II and proteoglycans (Figure 2). Co-delivery of IGF-1 and FGF-2 led to larger aggregate size, greater cellularity, and greater deposition of proteoglycan at days 14 and 28, as indicated by Safranin-O/fast green and toluidine blue, which displayed the spatial organization of the negatively charged proteoglycan with an orange to red hue and with a violet/red purple hue, respectively. Figure 2 shows the production of COL II (indicated by a prominent and uniform immunostaining in the aggregate) characteristic of cartilage matrix and indicator of chondrocyte-like phenotype; even a low but detectable immunostaining for COL I indicated a fibrous matrix related with ossification. The presence of COL X, a marker of hypertrophic chondrocytes undergoing terminal differentiation, was observed (Figure 2). A green tone was appreciated in the safranin-O staining at 28 days in the other groups tested, indicating that there is no detectable cartilage. Although phenotypic evidence of chondrogenesis was not greater in the other tested groups, the aggregates transduced with Ad.TGF-β1 showed a prominent inmmunostaining for COL II, primarily pericellular, and low immunostaining for COL I, making it an interesting candidate; it even showed a prominent immunostaining for COL X. No greater differences in immunostainings were noticed between the aggregates co-transduced with Ad.FGF-2 and Ad.IGF-1 and positive control. The qualitative nature of the test, however, did not allow proper statistical analysis.

### Time course of chondrocyte marker gene expression

To further examine the apparent synergistic effects of co-delivery of IGF-1 with FGF-2 in the aggregate cultures, the temporal expression profiles of genes associated with chondrogenic and osteogenic differentiation were analyzed. At days 3, 14, and 28, aggregates (*n *= 3) from each group (alone or in combination) were harvested, pooled, and analyzed using qRT-PCR (Figure 1B,C,D,E,F,G,H). Aggregate cultures of ASCs differentiated to chondrocytes by a commercial established medium and monolayer culture of newly transduced ASCs (time 0) were used as controls. Consistent with the preceding analyses, all of the aggregate cultures tested showed evidence of chondrogenic differentiation at the RNA level. The difference was seen from early time points, particularly at day 3, when the aggregates receiving Ad.FGF-2 and Ad.IGF-1 showed significant earlier onset of expression of AGC, BGC, CM, PGC and COL II with respect to the positive control (*P *= 0.012, *P *= 0.005, *P *<0.001, *P *= 0.006 and *P *= 0.958, respectively), and they maintained their significant values at high levels at all time points, being most remarkable at day 28. Even though the aggregates transduced with Ad.FGF-2/Ad.IGF-1 were also significant in their expression for COL I at day 3, this steadily decreased thereafter and was lower than in the other groups when expressed at day 28, excepting the aggregates transduced with Ad.FGF-2 alone. In addition, the expression of COL × mRNA in the Ad.IGF-1/Ad.FGF-2 group was very similar to the positive control with only greater expression at day 14 of culture.

**Figure 3:**
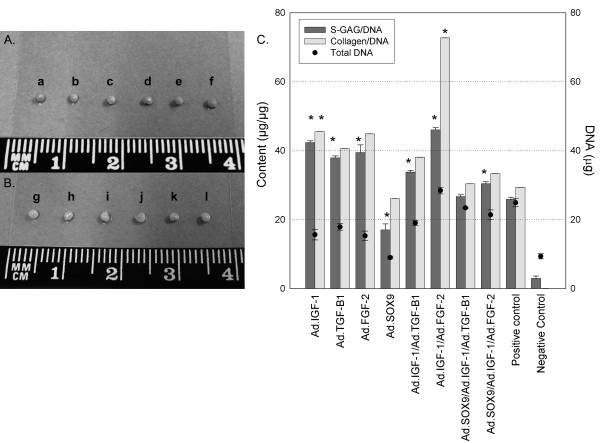
**Western blot and densitometry analysis**. **(A) **Western blot analyses at day 28 post transduction for *in vitro *expression of collagen (COL) I, COL II and COL X. **(B) **Densitometric analysis illustrating COL/glyceraldehyde-3-phosphate dehydrogenase (GAPDH) expression ratio (Phoretix 1D software; TotalLab Ltd, Newcastle, UK). Adipose-derived stem cells **(**ASCs) transduced with Ad.IGF-1/Ad.FGF-2 (multiplicity of infection 50 for each vector) showed almost threefold increased expression of COL II compared with the positive control. Similar low expressions of COL × were observed in adenoviral transduced ASCs and the positive control. Expression of COL I was undetected in the experimental groups. FGF-2, fibroblast growth factor-2; IGF-1, insulin-like growth factor-1; WB, positive control for type I collagen from cultured osteoblasts.

Other aggregate cultures that showed strong evidence of chondrogenic differentiation at the RNA level were those transduced with Ad.IGF-1 or co-transduced with Ad.IGF-1/Ad.TGF-β1, Ad.SOX9/Ad.IGF-1/Ad.TGF-β1, and Ad.SOX9/Ad.IGF-1/Ad.FGF-2. These groups showed not only a maintained high expression of AGC, BGC, CM, PGC and COL II, but also a markedly maintained high expression of COL I and COL X. To confirm immunohistochemistry and qRT-PCR results, western blot assays for COL I, COL II, and COL × detection in Ad.IGF-1/Ad.FGF-2 transduced ASCs at day 28 post transduction were performed. Densitometric analyses also demonstrated increased expression of COL II (threefold increased compared with the positive control), while expression of COL I and COL × was undetectable and limited, respectively (Figure 3). Answer: OK Our results suggest that IGF-1/FGF-2 co-expression accelerates and enhances the process of chondrogenesis.

### Biochemical assays for the content of DNA, glycosaminoglycans and collagen

**Figure 4:**
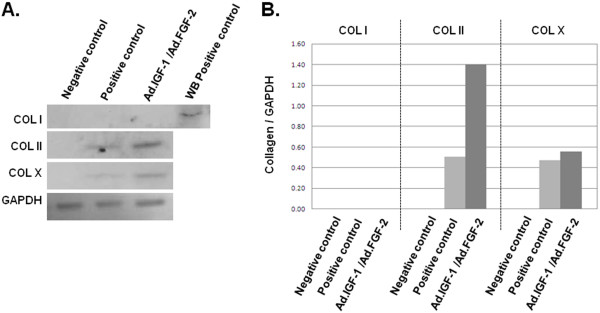
**Size and shape of aggregates and biochemical analyses**. Gross images of representative aggregates of each studied group are presented. **(A) **Aggregates transduced with single adenoviral vectors correspond to positive controls (a) and (b), Ad.SOX9 (c), Ad.FGF-2 (d), Ad.TGF-β1 (e), and AD.IGF-1 (f). **(B) **Aggregates transduced with combined adenoviral vectors correspond to positive controls (g) and (h), Ad.IGF-1/Ad.TGF-β1 (i), Ad.IGF-1/Ad.FGF-2 (j), Ad.SOX9/Ad.IGF-1/Ad.TGF-β1 (k) and, Ad.SOX9/Ad.IGF-1/Ad.FGF-2 (l). **(C) **Biochemical analyses of *in vitro *aggregates for total content of DNA, glycosaminoglycans (GAGs) and collagen. Aggregates were papain-digested and analyzed for total content of DNA, sulfated GAGs, and synthesized collagen. The content of GAGs and collagen were normalized by the DNA content of each sample. Data are presented as a mean ± standard deviation from three aggregates per group (*n *= 3). *P <0.05.

Representative aggregates from three aggregates per group are presented in Figure 4A,B. Aggregates co-transduced with Ad.IGF-1/Ad.FGF-2 were larger than the other groups and the positive control; even the aggregate transduced with Ad.IGF-1 was close to the millimeter size and was more uniform in shape. Evaluation of protein expression from individual and Ad.IGF-1/Ad.FGF-2 co-transduced aggregates showed a sustained transgene production at 14 and 28 days both alone and in combination (*P *>0.05). To quantitatively compare extracellular matrix synthesis between treatment groups, GAG and collagen levels in the aggregates after 28 days in culture were determined and normalized to DNA content. All aggregates co-transduced with Ad.IGF-1/Ad.FGF-2 showed significantly greater GAG and collagen production than the positive control (*P *<0.001). Furthermore, this group (Ad.IGF-1/Ad.FGF-2) showed more DNA (number of cells), GAG and collagen content (28.438 ± 0.943, 46.064 ± 0.587 and 72.744 ± 0.005, respectively) than the other groups (Figure 4C). Cultures transduced with Ad.GFP cultured in incomplete chondrogenic medium [24] did not form aggregates, and showed no phenotypic evidence of chondrogenesis. These findings correspond with the respective aggregate sizes and correlate with the respective histological and immunohistochemical findings (Figures 2 and 4A,B).

## Discussion

Previous studies have shown that primary MSCs undergo chondrogenesis after genetic modification with known chondrogenic factors as TGF-β2, bone morphogenic protein-2, IGF-1, and TGF-β1 and by induction of SOX9 expression in aggregate cultures *in vitro *and its application in articular cartilage repair *in vivo *[25-28]. In the present study, we adapted the ovine ASC aggregate culture system to determine whether adenoviral delivery of single and multiple growth and transcriptional factor genes can lead to efficient chondrogenesis *in vitro*.

We started by implementing some assays to characterize the ovine ASCs, since there are no commercially available reagents to study surface protein markers of this type of cell. Immunophenotype and qRT-PCR assays performed to first-passage of ASCs isolated showed high expression of mesenchymal stromal cell antigen-1, CD73, CD90, CD166, CD105, and CD271, low expression of CD14 and CD45, and lack of expression of CD34 and CD117, respectively. The low amplification of CD14 (considered a negative marker for ASCs) could be explained by the presence of other adherent cells (fibroblast, stromal, or monocytes), and/or lymphocytes and leucocytes not completely removed from the primary culture [29]. Immunophenotyping also showed a low percentage of CD45, which was decreasing along the subsequent passages as demonstrated by qRT-PCR assay (data not shown), a behavior that has been previously described in ASCs [30]. These results demonstrate successful ASC isolation and we report here a more complete ASC characterization method for this species.

Chondrogenesis differentiation of ASCs transduced with the different candidate growth and transcriptional factors was made using pellet culture to mimic the cellular condensation process during hyaline cartilage formation, with high spatial cell density and cell-cell contact, and is therefore often used as a method for understanding how the interaction of cells, growth factors, and environment promote a chondrogenic phenotype [24]. In this sense, we successfully optimized the traditional method for pellet culture preparation. To promote aggregate formation, we seeded the cells directly in a six-well plate, without trypsinizing the cells, and centrifuged them into 15 ml polypropylene conical tubes; we changed the medium for the corresponding chondrogenic medium and incubated the cells in appropriate conditions. After 3 days, spherical aggregates were formed alone. This modified method is easier, saves material and reagents, minimizes handling, and is compatible with ASC chondrogenesis.

In the present study, we demonstrate that combined overexpression of IGF-1 and FGF-2 within ASCs via adenoviral mediated-gene transfer significantly enhanced the chondrogenic differentiation after 28 days in an aggregate culture system *in vitro*, greater than with IGF-1, FGF-2, TGF-β1, SOX9 alone or in other combination. While previous studies have analyzed the effects of these factors on chondrogenesis using MSCs [6] or cartilage repair using chondrocytes [31,32], there has been no study assessing combination of all of these growth and transcriptional factors on chondrogenesis using ASCs.

The effects of growth factor co-expression on *in vitro *chondrogenesis of ASC aggregates through histologic examination indicated that aggregates receiving Ad.FGF-2 together with Ad.IGF-1 had greater matrix production than the other groups and control groups. The co-delivery of these growth factors led to larger aggregate size, greater cellularity, and greater deposition of proteoglycan. Although the production of COL II was prominent in the aggregates, the expression of COL × was also observed, suggesting the presence of hypertrophic chondrocytes undergoing terminal differentiation.

Aggregates transduced with TGF-β1 showed a prominent immunostaining for COL II, predominantly in the pericellular area, and low immunostaining for COL I, but they also showed increased expression of COL X. These findings are consistent with several previous studies of growth factor effects on MSC and ASC chondrogenesis, where TGF-β1 controls the production of extracellular matrices by stimulating the expression of AGC and collagens, and synthesis of COL II and COL X, which were secreted more strongly by MSCs than by ASCs [33,34].

Although the chondrogenic effects of TGF-β1 are well characterized, the effects of FGF-2 and IGF-1 are less well established. IGF-1 modulates MSC chondrogenesis by stimulating and increasing cell proliferation, regulates cell apoptosis, and induces *in vitro *expression of chondrocyte markers as proteoglycans and COL II [35,36]. IGF-1 has also been shown to improve chondrogenesis by increasing COL II and AGC expression when given in combination with TGF-β1, bone morphogenic protein-6 and TGF-β3 [12,37,38], but when administered alone is not sufficiently inductive in MSCs [39-41] or in ASCs, where exogenous protein was necessary in greater doses [42]. Our results show that IGF-1 not only stimulates the expression of chondrogenic markers, but also stimulates the expression of COL × in all the experimental groups in which it was tested. In monolayer cultures, FGF-2 increases cell proliferation, enhances the chondrogenic potential of MSCs, stabilizes phenotypic expression, and restrains terminal chondrocyte differentiation [43]. Mitogenic properties on *in vitro *articular chondrocytes have also been attributed to FGF-2. Enhancement of cartilage repair has been observed following the application of recombinant FGF-2 protein [44], transfected chondrocytes [45], or direct in gene transfer *in vivo *experiments using adeno-associated virus vectors into joint cartilage defects [46].

Our results show that FGF-2 not only stimulates the expression of chondrogenic markers, but also restrains the expression of COL I in all the experimental groups in which it was tested. A recent study has shown that combined overexpression of IGF-1 and FGF-2 within cartilage defects in alginate-embedded NIH 3T3 cells significantly enhances the repair of full-thickness osteochondral cartilage defects when compared with IGF-1 stimulation alone [13]. The study concluded that these two factors complement each other because FGF-2 enhances early chondrogenesis, whereas IGF-1 exerts its effects on chondrocyte proliferation and matrix synthesis at later time points. Despite the findings of this and other similar reports [47], the clear mechanism for chondrocyte differentiation exerted by IGF-1 and FGF-2 remains unclear. In our study, mRNA analyses for selected chondrocyte differentiation targets showed that aggregate culture with IGF-1 maintained high transcription of AGC, BGC, CM, PGC and COL II, but also showed a markedly significant maintained high expression for COL I and COL X. Cultured aggregates transduced with FGF-2 showed increased expression of BGC, CM, PGC and COL II, but decreased production of COL I and COL × through time. The aggregates receiving FGF-2 and IGF-1 showed significant earlier transcription of AGC, BGC, CM, PGC and COL II compared with the positive control, and expression of these markers was sustained at high levels at all time points, with most notable differences at day 28. Even though the aggregates transduced with Ad.FGF-2/Ad.IGF-1 also expressed COL I at day 3, expression of this protein decreased steadily thereafter and showed a nadir at day 28. In this group (Ad.IGF-1/Ad.FGF-2), the behavior of mRNA of COL × was very similar to the positive control with only greater expression at day 14 of culture.

The negative control group used in the gene expression analysis (Figure 1) showed endogenous basal expression for both the transduced genes (IGF-1, TGF-β1, FGF-2 and SOX9) and the cartilage-specific marker genes. Since cells in this group were cultured in incomplete chondrogenic medium without the induction of growth factors for 28 days, we assume that basal expression of these genes reflects their role in cell proliferation, survival, and involvement in an undetermined nonchondrogenic differentiation process.

Immunohistologic and western blot studies for this same experimental group of treatment resemble the mRNA expression behavior and clarify that there is an optimal production of COL II in 28-day cultured aggregates, while the presence of COL × and COL I is scarce and undetectable, respectively. There are suggestions that the expression of COL × should be considered with some caution; this protein has been regarded as a marker of hypertrophic differentiation, but Mwale and colleagues reported that COL × is expressed early during the process of chondrogenesis, even anticipating the production of COL II [48]. In conclusion, we demonstrate that a combination of IGF-1 and FGF-2 increases cell proliferation, GAG and collagen deposition, and renders acceptable results to create a predifferentiated implant of gene-modified ASC amenable for preclinical studies in the ovine model.

Limitations to this study include the use of only one sheep for experimental trials, which also was young, giving it greater regenerative capacity than older animals, and that this feature possible may mask the real functional contribution of the transduced genes in the chondrogenic differentiation capacity of ASCs. Additional analyses of combinations of selected factors not tested in this study are desirable to define the best transduction conditions for cartilage differentiation of ASCs, but the combination of IGF-1 and FGF-2 is amenable to starting preclinical studies in large animal models. These findings support the concept of implementing this gene transfer strategies for future research in articular cartilage repair.

## Conclusion

This study reports the enhanced chondrogenic differentiation of ASCs as a result of synergistically effect of combined overexpression of IGF-1/FGF-2 within ASCs by delivery adenoviral gene transfer, supported by analyses of gene expression, histological and biochemical as compared with the transduction of other known chondrogenic factors and transcriptions signals. We also found that this combination promotes significant production of COL II and other molecules involved in cartilage production (AGC, BGC, CM, and PGC), while it restrains the expression of COL I and COL X. The IGF-1/FGF-2 combination is amenable to generate an *in vitro *graft material for preclinical assays in large mammalian animal models for cartilage repair.

## Abbreviations

AGC: aggrecan; ASC: adipose-derived stem cell; BGC: biglycan; BSA: bovine serum albumin; CM: cartilage matrix; COL I: collagen I; COL II: collagen II; COL X: collagen X; DMEM: Dulbecco's modified Eagle's medium; ELISA: enzyme-linked immunosorbent assay; FBS: fetal bovine serum; FGF-2: fibroblast growth factor-2; GAG: glycosaminoglycan; GAPDH: glyceraldehyde-3-phosphate dehydrogenase; GFP: green fluorescent protein; IGF-1: insulin-like growth factor-1; mAb: monoclonal antibody; MOI: multiplicity of infection; MSC: mesenchymal stem cells; PBS: phosphate-buffered saline; PCR: polymerase chain reaction; PGC: proteoglycan; qRT: quantitative real time; RT: reverse transcriptase; SOX9: sex-determining region Y-box 9; TGFβ: transforming growth factor beta.

## Competing interests

The authors declare that they have no competing interests.

## Authors' contributions

IG-V carried out the isolation, culture, characterization and transduction of ASCs, qRT-PCR, biochemical assays, and statistical analysis. MG-R also participated in the qRT-PCR analyses. VJR-D and MAE-J carried out the histological and immunohistochemical analysis. IG-V and MLM-F carried out the construction of the recombinant adenoviral vectors and drafted the manuscript. IAM-M and DAB-G carried out the western blot and densitometry analysis. HGM-R and RO-L were involved in the design of the study and reviewed the manuscript. AR-M conceived of the study, and participated in its design and coordination and helped to draft the manuscript. The authors are responsible for the content and writing of this research paper. All authors read and approved the final manuscript.

## Supplementary Material

Additional File 1 table describing the primers used in the qRT-PCR analysis using the iScript™ One Step RT-PCR Kit with SYBR^® ^Green (Bio-Rad). F, forward (sense) primer; R, reverse (anti-sense) primer. Click here for file

Additional File 2 figure showing ASC viability with single and combined adenoviral transduction. Monolayers were transduced with increasing doses of Ad.GFP, Ad.IGF-1, Ad.TGF-β1, Ad.FGF-2 and Ad.SOX9 (A) alone and (B) in combination. At 10 days, cell viability was measured with the Alamar Blue assay; the optical density OD_570 to 600 nm _values of untransduced cells were set as 100%. Data expressed as mean standard error of triplicate experiments. (C) At 72 hours, GFP-positive cells were counted in three fields under light and fluorescence microscopy. Results are presented as the mean percentage of fluorescent cells per field at each viral dose. (D) Representative fluorescence of ASCs transduced with 1, 10, 100, and 1,000 MOIs of Ad.GFP, as indicated. Click here for file

Additional File 3 **figure showing Protein expression from ASC aggregates after adenoviral-mediated gene transfer of IGF-1 and FGF-2 alone and in combination**. Values represent levels of protein product (pg/ml) in (A,B,C) the conditioned medium at days 3, 7, 14, and 21, and (D,E,F) the aggregates at days 14 and 28. ASC aggregates singly infected with (A,D) Ad.IGF-1, (B,E) Ad.FGF-2, or (C,F) infected dually with Ad.IGF-1 at 50 MOIs and Ad.FGF-2 at 50 MOIs (100 MOIs together). Data represented as means standard deviations of three pellets per condition. Click here for file
