# Supplementary material for: Analyses of chondrogenic induction of adipose mesenchymal stem cells by combined co-stimulation mediated by adenoviral gene transfer
Source: Arthritis Res Ther. 2013 Jul 30;15(4):R80. doi: 10.1186/ar4260 (PMC3978573; doi:10.1186/ar4260)
Supplement: Additional File 1 — table describing the primers used in the qRT-PCR analysis using the iScript™ One Step RT-PCR Kit with SYBR® Green (Bio-Rad). F, forward (sense) primer; R, reverse (anti-sense) primer. [file ar4260-S1.PDF]

Table S1. Description of the primers used in this study.

Gene	Ref. no.	Primers 5’ → 3’	tm	PCR product (pb)
CD105	BC114687*	F: CTGATCCTCAGCGTGAACAA	60	226
		R: GACGAAGGAAGATGCTTTGC		
CD117	D16680.1*	F: CTCAGCCATCTGTGAGTCCA	60	191
		R: CTCTGCTTTCTCGGTGATCC		
CD14	NM_174008.1*	F: GCAGCCTGGAACAGTTTCTC	60	178
		R: TCCTCAAGCGTCAGTTCCTT		
CD166	NM_174238.1*	F: CGCAATGCAACAGGAGACTA	60	217
		R: GGCTAGATCGAAGCTTGACG		
CD271	BC142255.1*	F: CATCTCTGTGGACAGCCAGA	60	195
		R: TCAGGCTGGTAACCCAACTC		
CD34	NM_174009.1*	F: GGTGGCTGATACCGAACTGT	60	280
		R: TCAGCATCTTGGCTGTATGC		
CD45	BC148881.1*	F: CCACGGGTATTCAGCAAGTT	60	244
		R: CCCAGATCATCCTCCAGAAA		
CD73	NM_174129.3*	F: CTGAGACACCCGGATGAGAT	60	160
		R: ACTGGACCAGGTCAAAGGTG		
CD90	NM_001034765.1*	F: CACCTCTGCCAATACCACCT	60	196
		R: ATACCCCTCCATCCTTCCAC		
Aggrecan	NM_173981.2*	F: CAGAGTTCAGTGGGACAGCA	60	189
		R: AGACACCCAGCTCTCCTGAA		
Biglycan	BT021201.1*	F: ACCTCCCTGAGACCCTCAAT	60	184
		R: TTGTTGTCCAAGTGCAGCTC		
CatilageMatrix	X74326.1*	F: ATGCGGACAAGGTGGTAGAC	60	153
		R: TCTCCATACCCTGGTTGAGC		
ColII	X02420.1*	F: AGAGACCTGAACTGGGCAGA	60	211
		R: CAGAATAGCGCCGTTGTGTA		
ColI	FJ200442.1	F: GGTGACAGGAAGTCCCAGAA	60	167
		R: CCATCGTAGGTGACGCTGTA		
ColX	X53556.1*	F: ACCGAGAACGACCAGGTATG	60	279
		R: AACTCAAGAGGGCCTTCACA		
Proteoglycan	NM_174288.1*	F: TGCTGTGATTGCCTCTTTTG	60	169
		R: CCAAAACCCGTAGTTCCTGA		
FGF-2	NM_002006†	F: AGAAGAGCGACCCTCACATCA	60	237
		R: ACTGCCCAGTTCGTTTCAGTG		
IGF-1	NM_000618†	F: CAACAAGCCCACAGGGTATGG	60	219
		R: GCACTCCCTCTACTTGCGTTC		
TGF-β1	NM_000660†	F: GGCCAGATCCTGTCCAAGC	60	201
		R: GTGGGTTTCCACCATTAGCAC		
SOX9	NM_000346†	F: AGACAGCCCCCTATCGACTTC	60	230
		R: TGCTGCTTGGACATCCACAC		
GAPDH	AF022183.1	F: GATTGTCAGCAATGCCTCCT	60	194
		R: AAGCAGGGATGATGTTTTGG		

*Sequence designed based on *Bos Taurus* genome due to *Ovis aries* genome is not ensembled for that gen.
†Sequence designed based on human genome.
