# Supplementary figures and images for: Analyses of chondrogenic induction of adipose mesenchymal stem cells by combined co-stimulation mediated by adenoviral gene transfer
Source: Arthritis Res Ther. 2013 Jul 30;15(4):R80. doi: 10.1186/ar4260 (PMC3978573; doi:10.1186/ar4260)

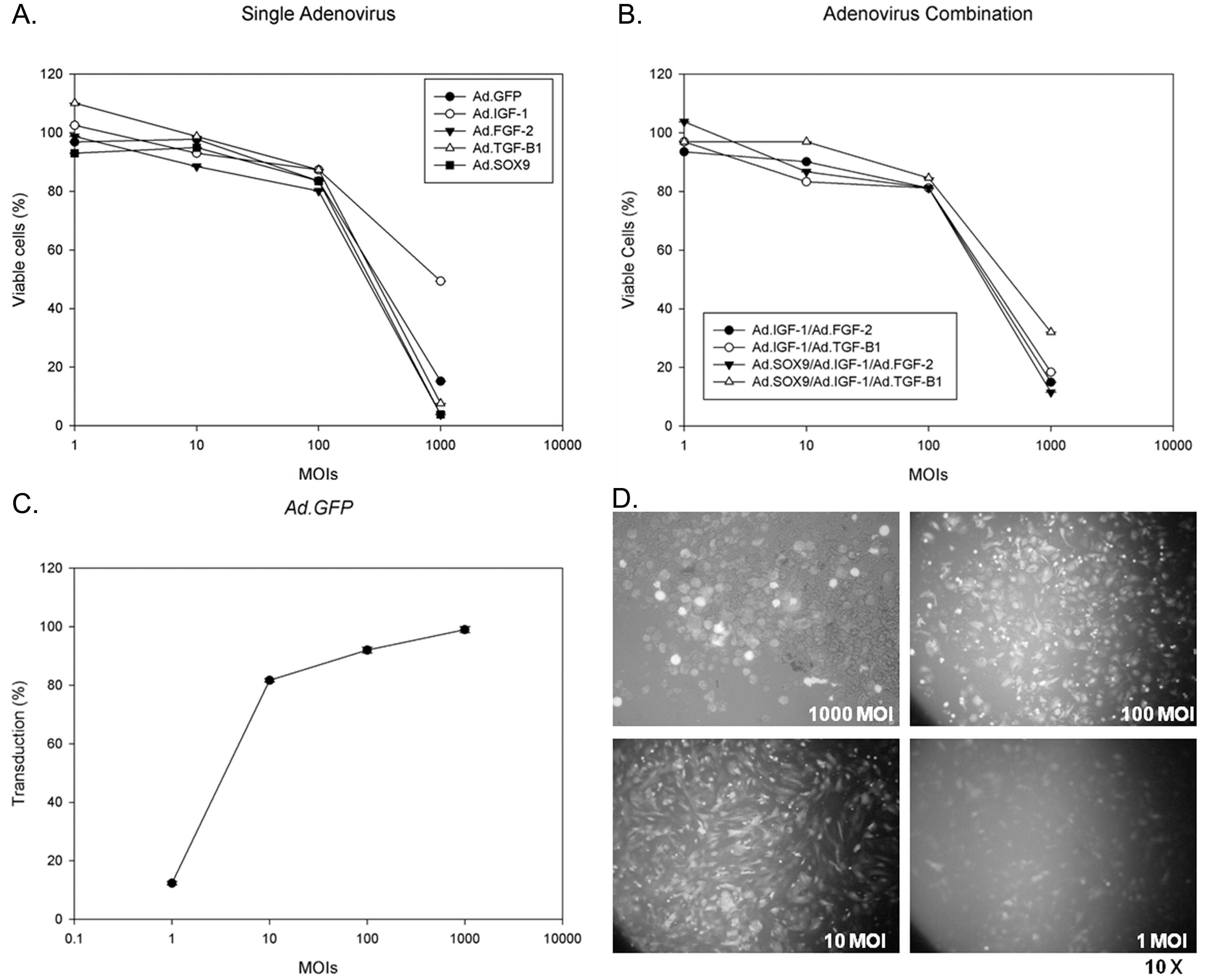

Supplement: Additional File 2 — figure showing ASC viability with single and combined adenoviral transduction. Monolayers were transduced with increasing doses of Ad.GFP, Ad.IGF-1, Ad.TGF-β1, Ad.FGF-2 and Ad.SOX9 (A) alone and (B) in combination. At 10 days, cell viability was measured with the Alamar Blue assay; the optical density OD570 to 600 nm values of untransduced cells were set as 100%. Data expressed as mean standard error of triplicate experiments. (C) At 72 hours, GFP-positive cells were counted in three fields under light and fluorescence microscopy. Results are presented as the mean percentage of fluorescent cells per field at each viral dose. (D) Representative fluorescence of ASCs transduced with 1, 10, 100, and 1,000 MOIs of Ad.GFP, as indicated. [file ar4260-S2.JPEG]

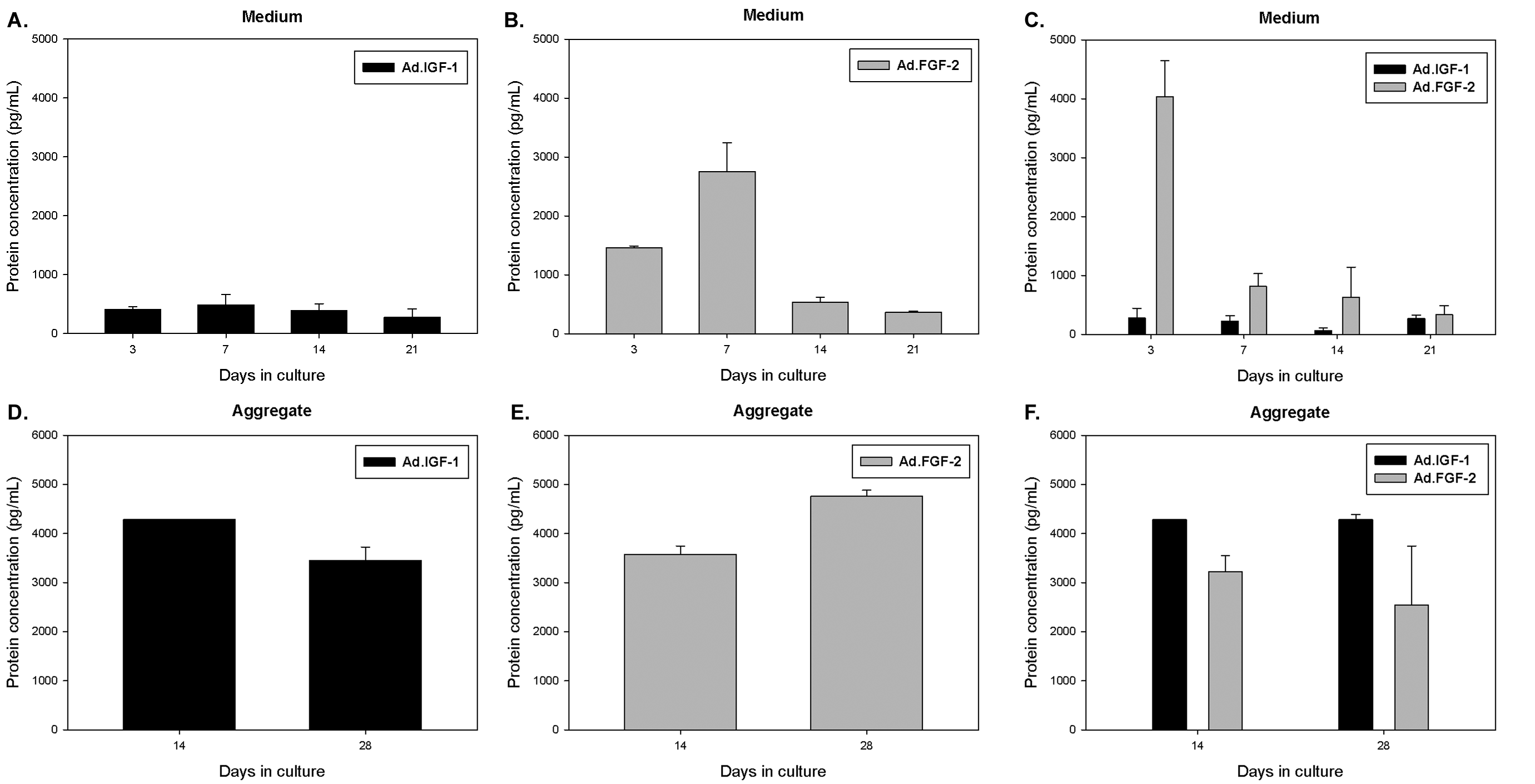

Supplement: Additional File 3 — figure showing Protein expression from ASC aggregates after adenoviral-mediated gene transfer of IGF-1 and FGF-2 alone and in combination. Values represent levels of protein product (pg/ml) in (A,B,C) the conditioned medium at days 3, 7, 14, and 21, and (D,E,F) the aggregates at days 14 and 28. ASC aggregates singly infected with (A,D) Ad.IGF-1, (B,E) Ad.FGF-2, or (C,F) infected dually with Ad.IGF-1 at 50 MOIs and Ad.FGF-2 at 50 MOIs (100 MOIs together). Data represented as means standard deviations of three pellets per condition. [file ar4260-S3.TIFF]
